# Phenome-wide association study identifies new clinical phenotypes associated with *Staphylococcus aureus* infections

**DOI:** 10.1371/journal.pone.0303395

**Published:** 2024-07-05

**Authors:** Patrick Allaire, Noha S. Elsayed, Richard L. Berg, Warren Rose, Sanjay K. Shukla

**Affiliations:** 1 Center for Precision Medicine Research, Marshfield Clinic Research Institute, Marshfield, Wisconsin, United States of America; 2 Research Computing and Analytics, Marshfield Clinic Research Institute, Marshfield, Wisconsin, United States of America; 3 School of Pharmacy, University of Wisconsin, Madison, Wisconsin, United States of America; 4 Computational and Informatics in Biology and Medicine Program, University of Wisconsin-Madison, Madison, Wisconsin, United States of America; University of Calgary, CANADA

## Abstract

**Background:**

**Phe**nome-**W**ide **A**ssociation study (**PheWAS**) is a powerful tool designed to systematically screen clinical observations derived from medical records (phenotypes) for association with a variable of interest. Despite their usefulness, no systematic screening of phenotypes associated with ***S****taphylococcus* ***a****ureus* **i**nfection**s** (SAIs) has been done leaving potential novel risk factors or complications undiscovered.

**Method and cohorts:**

We tailored the PheWAS approach into a two-stage screening procedure to identify novel phenotypes correlating with SAIs. The first stage screened for co-occurrence of SAIs with other phenotypes within medical records. In the second stage, significant findings were examined for the correlations between their age of onset with that of SAIs. The PheWAS was implemented using the medical records of 754,401 patients from the Marshfield Clinic Health System. Any novel associations discovered were subsequently validated using datasets from TriNetX and *All of Us*, encompassing 109,884,571 and 118,538 patients respectively.

**Results:**

Forty-one phenotypes met the significance criteria of a p-value < 3.64e-5 and odds ratios of > 5. Out of these, we classified 23 associations either as risk factors or as complications of SAIs. Three novel associations were discovered and classified either as a risk (long-term use of aspirin) or complications (iron deficiency anemia and anemia of chronic disease). All novel associations were replicated in the TriNetX cohort. In the *All of Us* cohort, anemia of chronic disease was replicated according to our significance criteria.

**Conclusions:**

The PheWAS of SAIs expands our understanding of SAIs interacting phenotypes. Additionally, the novel two-stage PheWAS approach developed in this study can be applied to examine other disease-disease interactions of interest. Due to the possibility of bias inherent in observational data, the findings of this study require further investigation.

## Introduction

Although the Electronic Health Record (EHR) became mainstream in the United States’ healthcare systems in the early 2000s, research using these databases had been somewhat limited in scope. In 2010, a seminal Phenome-Wide Association Study (PheWAS) found that EHR data (e.g., a phenotype) can be screened against a genetic variant to replicate known genomic associations [1]. This has helped understand genetic variant associated phenotypic pleiotropy. Using this PheWAS paradigm, the genetic variant can be replaced by any other variable of interest, for instance clinical phenotypes presented by *Staphylococcus aureus* infections (SAIs). Using large cohorts with rich longitudinal EHR data, this adaptation of the PheWAS was a powerful opportunity to identify the spectrum of clinical phenotypes associated with SAIs like what has been shown with COVID-19 [2].

Diseases such as bacteremia, endocarditis, and osteomyelitis resulting from *S*. *aureus* cause significant morbidity and mortality [3,4]. SAIs (referring to all *S*. *aureus* causative diseases) pose a major problem for both inpatient and outpatient settings [5]. For example, the incidence rate of *S*. *aureus* bacteremia is up to 65 cases/100,000 patients/year [6]. SAI’s consequences include high mortality, prolonged hospitalization, and excessive healthcare costs [7]. In a 2010–2014 study, both MRSA (methicillin-resistant *S*. *aureus*) and MSSA (methicillin-sensitive *S*. *aureus*) led to excessive hospitalization costs [8]. Risk factors for SAIs include prolonged hospitalization, surgical procedures, immunocompromised status, type 2 diabetes, and glucocorticoid treatment [9–12]. An effective management of SAIs cannot be accomplished without a full understanding of all known and yet-to-be-known disease risk factors and downstream disease complications [13]. Given the intricacies inherent in studying patients with SAIs and the sizeable amount data available in modern EHR system, larger and more comprehensive studies can be done, which increases the opportunity to find novel associations with SAI. This, in turn, may improve our understanding of the SAIs.

To explore the SAIs-phenome interaction spectrum, we implemented a two-step PheWAS to identify novel associations using EHR data from a multispecialty Marshfield Clinic Health System (MCHS) in Marshfield, WI. Among many associations found, we identified three new phenotypes associated with SAIs and these associations were reproduced in datasets from the *All of Us*, a National Institutes of Health Research database and TriNetX, a global health research network.

## Materials and methods

### Ethic statement—Human subject research

This study utilized data from three cohorts: MCHS, TriNetX, and *All of Us* Research Project (*AoU*RP). The data from these cohorts was previously de-identified. The authors had no access to any type of data that can potentially identify participants, except for ICD code dates required to establish time correlations between two diseases. This manuscript neither discusses individual-level data nor gives exact group size numbers for those smaller than 20 individuals.

**MCHS:** The research contained in this article was approved by the institutional review board of the Marshfield Clinic Research Institute, IRB # IRB-18-056 was granted to Sanjay K Shukla Ph.D. on December 12^th^, 2021 for study number SHU10614. Informed Consent was not required as determined by the MCHS IRB as all the data were analyzed anonymously.
**TriNetX:** The utilization of data from the TriNetX platform was exempted from requiring ethical approval at the researcher level. This exemption is due to the thorough de-identification process the data undergoes, which has been certified as HIPAA-compliant through expert determination. Since both TriNetX and the *All of Us* data are de-identified, and the research did not involve any intervention or interaction with living individuals, it is classified as "Not Human Subject" research. For more details on the TriNetX de-identification process, refer to: https://trinetx.com/wp-content/uploads/2022/04/TriNetX-Empirical-Summary-by-Brad-Malin-2020_branded.pdf
***All of Us* Research Program**: Similarly, the use of data from the *All of Us* Research Program doesn’t require ethical approval at the researcher level, thanks to its comprehensive de-identification procedure. While *All of Us* has specific criteria, such as studying groups smaller than 20, which would necessitate an IRB approval, our study doesn’t fall within these parameters. More about the *All of Us* Research Program’s IRB-approved protocol can be accessed here: https://allofus.nih.gov/about/all-us-research-program-protocol

### Study cohorts description

The first, discovery cohort consisted of EHR from 754,401 patients form MCHS. Our inclusion criteria included a minimum of 18 years of age and at least 5 years of EHR data defined by any ICD code entry on two separate days. We extracted the dataset for this cohort once on March 20^th^, 2022. The second cohort, TriNetX, consisted of 82 healthcare organizations for a combined EHR data from 109,884,571 patients. We accessed this data directly from the TriNetX platform on of May 15^th^, 2023 and used it to calculate odd ratios (see below). Note that due to limitation of TriNetX data access, the initial inclusion criteria for this cohort were only to have all patients be at least 18 years of age with no restriction with length of EHR. To investigate the correlation of age of onset between SAI and other phenotypes, a sub-TriNetX cohort that included only patients with SAI was downloaded by us and included their full, de-identified, EHR records. This allowed to restrict patient eligibility as performed above for the MCHS cohort. This dataset was obtained on March 28^th^, 2022. The third cohort was derived from *All of Us* [14] and consisted of 118,538 participants filtered from 413,457 initial participant entries (CDR version C2022Q4R9, May 2023). Of the 413,457 participants, 287,012 had some type of EHR data accessible, and 254,487 have EHR defined by any ICD code entry on two separate days. We restricted the parameters more tightly to participants with five or more years EHR data result. That gave us a cohort of 166,790 individuals, of whom 118,538 had genetically predicted ancestry; these we retained. The TriNetX research network and *All of Us* cohort were used as a replication cohort for any novel SAIs associations identified with the MCHS cohort. Demographics of the cohorts used in the first step PheWAS are presented in S1.1 Table in S1 Table and those for the second step PheWAS in S1.2 Table in S1 File.

### Two steps PheWAS screen

**Fig 1:**
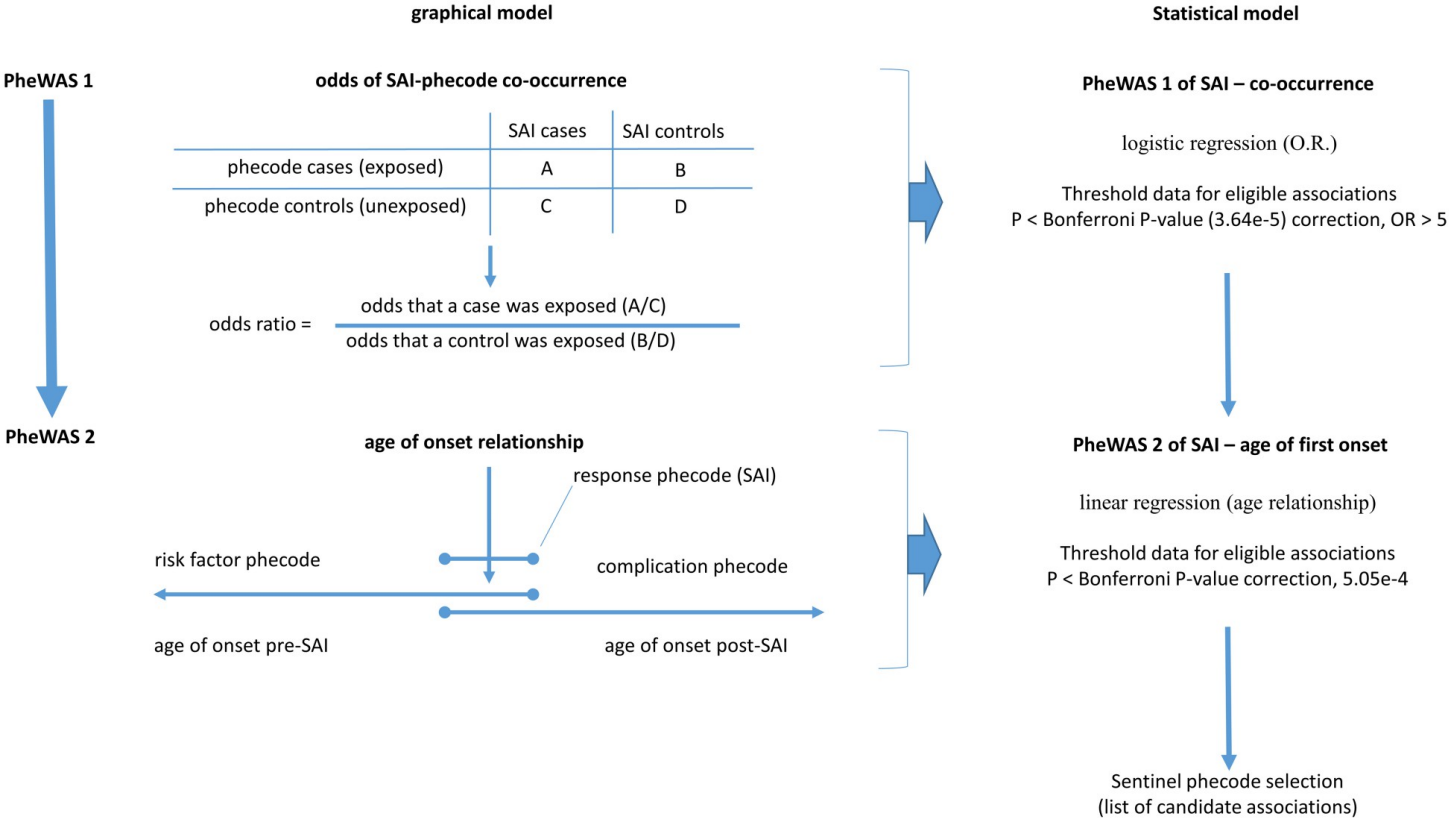
Two step PheWAS screening approach. Flow chart of the PheWAS 1 and 2 screening approaches and steps used in PheWAS 1 and PheWAS 2 statistical model.

The two steps PheWAS screening was conducted with the MCHS dataset, as outlined in Fig 1, to identify novel associations. The dual PheWAS approach utilizes different input parameters to evaluate associations between SAIs and a phecode (codes that rapidly define case/control status of hundreds of clinical conditions). Briefly, a disease in EHR is defined by ICD-9 and ICD-10 codes. These ICD codes are mapped to 1866 phecodes (extracted from phecode map version 1.2; https://phewascatalog.org/phecodes) [15] for use in the PheWAS. In the first PheWAS, we screened medical records for phecodes that coincided with SAIs phecode, 41.1. We chose Phecode 41.1 as it encompasses all forms of methicillin-sensitive or methicillin-resistant SAIs at any clinical site including blood. Phecode 41.1 also includes the history of the disease as stated elsewhere. Here, a logistic regression model was used where the response variable was SAIs, and the other phenotype was the predictor. The model included age at the last healthcare visit, sex, and ancestry (or race) as covariates. Note that race was used instead of ethnicity in TriNetX as the latter was not available. Basic cohort characteristics for this PheWAS are given in S1.1 Table in S1 Table. Given that SAIs are definitive diagnoses, we only required one occurrence of a phecode to designate a case. Those patients without a SAIs phecode record were labelled as controls. We considered association results only when at least 50 patients were coded for both SAIs and the test phecode [16]. Although we acknowledge the subjective nature of this minimum count, the goal here was to screen the sample size and remove imprecise estimates from consideration.

This screening left 1373 phecodes tested. All those phecodes which reached Bonferroni adjusted p-value threshold for multiple testing (p<3.64e-5, alpha = 0.05, 1373 tests) and with an odds ratio (OR) of ≥ 5 were carried into the second sequential PheWAS. The high OR selection, which included those over the 75% percentile, reflects our goal to focus only on the most impactful associations, although we understand that less significant but relevant association may be missed.

The second PheWAS tested the relation of age at first onset between SAIs and a phecode. To avoid statistical inflation caused by one-time visits that could lead to aberrant simultaneous coding of SAIs and any other phecode, we excluded any patient where the onsets of SAIs and the phecode were within 24 h (1 day). Age at first SAIs (defined as the first ICD code entry mapped to SAI) was assigned as the response variable and onset of the test phenotype (defined as the first ICD code entry mapped to this phenotype) as a predictor variable. Covariates for adjustment included age, sex, and race at the last visit. Basic cohort characteristics for this PheWAS are given in S1.2 Table in S1 File. In this second screen, we considered only association test results where at least 50 patients presented the phecode within 60 days of onset of SAIs. We retained phecodes reaching Bonferroni adjusted p-value threshold for multiple testing (p<5.05e-4, alpha = 0.05, 99 tests) for further classification either as risk factors or complications of a SAI, based on which condition appeared first.

Next, we removed patients with known risk factors, including: phecodes 197 (chemotherapy), 250 (all type of diabetes), 429.1 (heart transplant/surgery), 510.2 (lung transplant), 573.2 (liver replacement by transplant), 585.32 (end stage renal disease), 851 (complications of transplant and reattached limbs), and 860 (bone marrow or stem cell transplant). Once this removal was complete, we performed the second PheWAS. This order helped account for known risk factors. Statistical models are formally defined in S2 Table. All statistical analyses were performed in R, version 3.6.3 or higher and using routine basic packages.

### Replication of novel associations with TriNetX and *All of Us*

In Table 3, we calculated odds ratios (ORs) using a contingency table because TriNetX web-based analysis platform limits access to all TriNetX EHR data simultaneously. Therefore, these ORs are not adjusted for age, sex, and race. To make for a fair comparison, we also recalculated MCHS ORs using a contingency table and data derived from TriNetX (which includes MCHS) to develop two comparable datasets. The PheWAS of age of first onset uses linear regression models and were performed as described above. Results for *All of Us* are as detailed as was for TriNetX.

### Redundant phecode pruning

Since phecodes within a class are often biologically similar, reporting the whole class may provide redundant information. To limit redundancy, we selected sentinel phenotype for each class of phecodes as the one with the highest ORs. For example, phecode class 250 represents both types of diabetes whereas the biology of type I diabetes (T1D) is different from type II diabetes (T2D) and we made that distinction in sentinel phecode selection. The sub-class phecode 250.1 represents T1D and has six sub-phecodes (250.10 to 250.15). The sub-class phecode 250.2 represents T2D and has also 6 sub-phecodes (250.20 to 250.15). We considered only sub-phecodes with p-value that met the Bonferroni threshold, and within those codes, chose the one with the highest OR as the sentinel phecode. Using T1D as an example, phecodes 250.10 and 250.13 were significant but 250.13 had a higher OR than 250.10. Therefore, only 250.13 was reported and represented the sentinel phecode of that group. A similar approach was used for T2D. If there were no obvious underlying biological distinctions within a phecode class, which was true for most classes, all sub-codes were curated together.

## Results

The MCHS cohort, which was utilized as the discovery cohort, consisted of 754,401 patients, 52.4% of whom were female. The average age of the cohort was 55.6 years (SD = 20.7), with an average EHR length of 20.5 (SD = 10.2) years. It is notable that a significant proportion of the MCHS patient population (61.1%) self-identified as white of European descent. However, the ethnicity of a considerable fraction (36.7%) of this EHR population remained unknown (S1.1 Table in S1 Table).

### The two steps PheWAS screen identified 41 phecodes associated with SAIs

The two-step PheWAS process with a threshold for flagging associations is illustrated in Fig 1, with details of the individual PheWAS model described in S2 Table. All statistical models were adjusted for age and sex at the last diagnosis. The only exception to this was the manual calculation of ORs performed with TriNetX and the equivalent for MCHS and *AoU*RP in Table 3 (see Methods). The first PheWAS identified 236 associations (S3.1 Table in S3 Table), and these were carried forward to the second PheWAS for age correlation. Due to our criteria, which required at least 50 patients to have their first phecode registered within 60 days of the first SAI recorded, and the removal of individuals coded within 24 hours of an SAI (to avoid potential data inflation from one-visit specialty treatments), only 99 out of the 236 phecodes were eligible for testing in the second PheWAS. After the second screening, 94 phecodes remained significant (S4.1 Table in S4 Table). However, many of these phecodes belonged to the same phecode class, rendering them biologically similar and redundant. To address this, we selected the top association within each phecode class based on its p-value significance and the highest OR (see Materials and methods and S5 Table). We referred to this top association as the ’sentinel phecode’. This selection process reduced the number of significant associations to 41. The summary statistics from the two-step PheWAS for these 41 phecodes are presented in Table 1.

**Table 1:** Summary statistics of MCHS dual phewas of sais (sentinels).

Phecode	Phenotype	PheWAS Occurrence			PheWAS Age of First Onset			Disease Category	Known Association
		O.R.	S.E.	p-value	beta	S.E.	p-value		
401.21	Hypertensive heart disease	6.062	1.038	0.00E+00	0.144	0.023	3.43E-10	circulatory system	yes
420.3	Endocarditis	7.271	1.057	1.68E-276	0.293	0.037	1.33E-14	circulatory system	yes
427.7	Tachycardia NOS	5.962	1.034	0.00E+00	0.461	0.033	2.15E-41	circulatory system	yes
428.1	Congestive heart failure (CHF) NOS	7.412	1.035	0.00E+00	0.292	0.028	2.13E-25	circulatory system	yes
452.2	Deep vein thrombosis [DVT]	5.025	1.043	0.00E+00	0.235	0.021	2.75E-27	circulatory system	yes
457.3	Encounter for long-term (current) use of aspirin	6.502	1.031	0.00E+00	0.19	0.011	2.20E-61	circulatory system	**No**
459.1	Hemorrhage NOS	7.741	1.073	8.13E-186	0.521	0.07	9.89E-13	circulatory system	yes
681.2	Cellulitis and abscess of face/neck	9.353	1.054	0.00E+00	0.117	0.022	1.19E-07	dermatologic	yes
686.1	Carbuncle and furuncle	9.008	1.048	0.00E+00	0.054	0.012	9.23E-06	dermatologic	yes
707.1	Decubitus ulcer	12.625	1.037	0.00E+00	0.276	0.016	1.07E-64	dermatologic	yes
250.25	Diabetes type 2 with peripheral circulatory disorders	7.099	1.04	0.00E+00	0.239	0.028	2.35E-17	endocrine/metabolic	yes
275.3	Disorders of magnesium metabolism	6.721	1.033	0.00E+00	0.197	0.014	7.94E-44	endocrine/metabolic	yes
276.11	Hyperosmolality and/or hypernatremia	8.031	1.058	1.32E-296	0.483	0.064	2.15E-13	endocrine/metabolic	yes
580.3	Nephritis and nephropathy, no mention of glomerulonephritis	5.133	1.051	1.13E-240	0.069	0.015	3.66E-06	genitourinary	yes
585.1	Acute renal failure	8.344	1.031	0.00E+00	0.195	0.011	6.71E-67	genitourinary	yes
592.11	Acute cystitis	5.318	1.044	0.00E+00	0.239	0.046	2.63E-07	genitourinary	yes
599.2	Retention of urine	5.227	1.038	0.00E+00	0.07	0.013	8.65E-08	genitourinary	yes
280.2	Iron deficiency anemia secondary to blood loss (chronic)	6.216	1.055	1.31E-257	0.403	0.055	8.26E-13	hematopoietic	**No**
285.2	Anemia of chronic disease	6.825	1.035	0.00E+00	0.216	0.013	1.72E-54	hematopoietic	**No**
286.9	Abnormal coagulation profile	6.174	1.038	0.00E+00	0.181	0.015	2.46E-31	hematopoietic	yes
288.2	Elevated white blood cell count	6.989	1.035	0.00E+00	0.33	0.027	3.18E-32	hematopoietic	yes
8.52	Intestinal infection due to C. difficile	8.774	1.069	1.72E-230	0.671	0.063	8.21E-23	infectious diseases	yes
38.2	Gram positive septicemia	116.252	1.066	0.00E+00	0.855	0.04	6.00E-76	infectious diseases	yes
112.3	Candidiasis of skin and nails	6.206	1.05	5.44E-303	0.081	0.013	1.60E-09	infectious diseases	yes
964.1	Anticoagulants causing adverse effects	5.763	1.068	1.92E-157	0.112	0.024	3.18E-06	injuries & poisonings	yes
994.21	Septic shock	11.206	1.049	0.00E+00	0.338	0.025	7.14E-38	injuries & poisonings	yes
290.2	Delirium due to conditions classified elsewhere	6.685	1.065	1.54E-202	0.158	0.03	2.24E-07	mental disorders	yes
292.4	Altered mental status	6.658	1.032	0.00E+00	0.227	0.015	2.50E-50	mental disorders	yes
710.11	Acute osteomyelitis	26.273	1.047	0.00E+00	0.232	0.02	1.45E-29	musculoskeletal	yes
711.1	Pyogenic arthritis	21.78	1.053	0.00E+00	0.114	0.019	6.49E-09	musculoskeletal	yes
741.3	Difficulty in walking	5.082	1.037	0.00E+00	0.141	0.018	1.45E-14	musculoskeletal	yes
348.8	Encephalopathy, not elsewhere classified	7.855	1.04	0.00E+00	0.135	0.017	7.85E-15	neurological	yes
355.1	Chronic pain syndrome	6.258	1.042	0.00E+00	0.212	0.021	1.70E-22	neurological	yes
480.13	MRSA pneumonia	24.262	1.132	1.44E-146	0.235	0.038	9.92E-09	respiratory	yes
509.8	Dependence on respirator or supplemental oxygen	6.054	1.036	0.00E+00	0.237	0.023	9.69E-25	respiratory	yes
512.7	Shortness of breath	5.32	1.028	0.00E+00	0.091	0.006	3.26E-47	respiratory	yes
771.1	Swelling of limb	6.447	1.028	0.00E+00	0.075	0.007	4.27E-30	symptoms	yes
772.3	Muscle weakness	5.921	1.032	0.00E+00	0.258	0.017	3.42E-51	symptoms	yes
782.3	Edema	5.624	1.029	0.00E+00	0.061	0.006	1.34E-26	symptoms	yes
790.8	Elevated C-reactive protein (CRP)	5.81	1.06	1.17E-198	0.249	0.029	6.93E-17	symptoms	yes

O.R.: Odds Ratio

S.E.: Standard Error

### Sentinel phecodes associated with SAIs enriches in circulatory system disease category

We classified phecodes into disease categories to give a general sense of their association with biological/physiological pathways [17], (https://phewascatalog.org/). Out of 17 disease categories, 12 phecodes were found to be associated with SAIs (S6 Table). An enrichment of circulatory system phecodes emerged (7/41, or 17% of total phecodes), indicating that endovascular physiology is strategically important for SAIs. This is further supported by the inclusion of five other phecodes categorized under the hematopoietic category, which interacts with the circulatory system through the blood transport of hematopoietic cells. Altogether, these observations suggest that a dysfunctional blood/circulatory system may either provide an opportunity for infection, or that SAIs can perturb the system.

### Phecodes associated with SAIs are mainly risk factors

To further unravel how the 41 phecodes were associated with SAIs, we classified them as either a potential risk factor or a complication of SAIs. We counted the number of times the age of first onset of a phecode occurred before (therefore a risk) SAIs versus after (a complication). We counted occurrences (pre-SAIs and post-SAIs) for both lifetime and acute, which we defined as within 60 days of a SAIs onset (Table 2). Using this classification method, out of the 41 phecodes, 24 associations were classified as either risk factors or complications of SAIs, and 17 remained unclassified (referred to as cryptic). Not surprisingly, some clinical identifiers of SAIs appeared as risk factors and represent 17 out of the 23 as shown in Table 2. We carried out a search through both PubMed and Google to identify established or reported associations in the literature. Criteria for labeling an association as established were the presence of more than one confirmatory study or a large patient cohort or a finding with an odds ratio of >2. Criteria for previously reported but less than established associations were as: i) mentioned in a case study or, ii) results from in vitro experimentation, and/or iii) symptoms of established associations like shortness of breath for MRSA pneumonia (Table 2).

**Table 2:** Phenotype risk versus complication. Ratio = counts of risk / counts of complication.

Phenotype	Lifetime Onset Counts				Acute Onset Counts							
	Total	Risk	Compl.	Ratio	Total	Risk	Compl.	Ratio	Category	References	Ratio const.	Interpretation
Iron deficiency anemia secondary to blood loss (chronic)	541	223	318	**0.7**	110	42	68	**0.62**	Hematopoietic	Not reported yet	Complication	**New complication**
Hypertensive heart disease	1368	710	658	**1.08**	175	101	74	**1.36**	Circulatory system	Not reported yet	Risk	Clinical identifier****
Congestive heart failure	1914	1053	861	**1.22**	263	133	130	**1.02**	Circulatory system	[38,39]*	Risk	Known risk factor
Encounter for long-term (current) use of aspirin	3422	2700	722	**3.74**	298	185	113	**1.64**	Circulatory system	Not reported yet	Risk	**New risk factor**
Cellulitis and abscess of face/neck	568	354	214	**1.65**	89	69	20	**3.45**	Dermatologic	[40–42]*	Risk	Clinical identifier
Carbuncle and furuncle	951	735	216	**3.4**	100	72	28	**2.57**	Dermatologic	[43–45]*	Risk	Clinical identifier
Decubitus ulcer	1630	1093	537	**2.04**	297	178	119	**1.5**	Dermatologic	[23,25]*	Risk	Clinical identifier
Disorders of magnesium metabolism	1962	1375	587	**2.34**	246	153	93	**1.65**	Endocrine/Metabolic	[22]**	Risk	Clinical identifier
Acute renal failure	2849	2119	730	**2.9**	411	274	137	**2**	Genitourinary	[46,47]*	Risk	Known risk factor
Retention of urine	1331	836	495	**1.69**	230	152	78	**1.95**	Genitourinary	**	Risk	Known risk factor
Abnormal coagulation profile	1452	997	455	**2.19**	167	102	65	**1.57**	Hematopoietic	[48]**	Risk	Clinical identifier
Elevated white blood cell count	1465	747	718	**1.04**	296	162	134	**1.21**	Hematopoietic	[20]**	Risk	Clinical identifier
Anticoagulants causing adverse effects	484	330	154	**2.14**	52	27	25	**1.08**	Injuries & poisonings	[48]**	Risk	Clinical identifier
Septic shock	705	383	322	**1.19**	160	106	54	**1.96**	Injuries & poisonings	[49]*	Risk	Clinical identifier
Delirium due to conditions classified elsewhere	448	232	216	**1.07**	82	49	33	**1.48**	Mental disorders	[21]**	Risk	Clinical identifier
Altered mental status	2259	1386	873	**1.59**	365	201	164	**1.23**	Mental disorders	[50,51] *	Risk	Clinical identifier
Pyogenic arthritis	513	344	169	**2.04**	140	87	53	**1.64**	Musculoskeletal	[28,52]*	Risk	Clinical identifier
MRSA pneumonia	117	84	33	**2.55**	53	36	17	**2.12**	Respiratory	[27,53]*	Risk	Clinical identifier
Dependence on respirator [ventilator] or supplemental oxygen	1458	760	698	**1.09**	233	117	116	**1.01**	Respiratory	[27,53]**	Risk	Clinical identifier
Shortness of breath	5331	4303	1028	**4.19**	389	226	163	**1.39**	Respiratory	[27,53]**	Risk	Clinical identifier
Swelling of limb	3840	3044	796	**3.82**	354	198	156	**1.27**	Symptoms	[54]**	Risk	Clinical identifier
Edema	4377	3627	750	**4.84**	351	191	160	**1.19**	Symptoms	[55]**	Risk	Clinical identifier
Endocarditis	459	224	235	**0.95**	132	66	66	**1**	Circulatory system	[19,37]*	Cryptic*	N/A
Tachycardia	1508	712	796	**0.89**	291	156	135	**1.16**	Circulatory system	[56]**	Cryptic	N/A
Deep vein thrombosis [DVT]	995	659	336	**1.96**	149	74	75	**0.99**	Circulatory system	[57,58]*	Cryptic	N/A
Hemorrhage	327	137	190	**0.72**	58	30	28	**1.07**	Circulatory system	[59]**	Cryptic	N/A
Hyperosmolality and/or hypernatremia	471	197	274	**0.72**	128	79	49	**1.61**	Endocrine/Metabolic	[60–62]*	Cryptic	N/A
Nephritis and nephropathy, no mention of glomerulonephritis	739	595	144	**4.13**	55	26	29	**0.9**	Genitourinary	N/A	Cryptic	N/A
Acute cystitis	989	437	552	**0.79**	127	71	56	**1.27**	Genitourinary	Not reported yet	Cryptic	N/A
Anemia of chronic disease	1710	1135	575	**1.97**	256	120	136	**0.88**	Hematopoietic	New	Cryptic	**N/A**
Intestinal infection due to C. *difficile*	330	128	202	**0.63**	74	42	32	**1.31**	Infectious diseases	[63]**	Cryptic	N/A
Gram positive septicemia	583	272	311	**0.87**	374	229	145	**1.58**	Infectious diseases	[49]*	Cryptic	N/A
Candidiasis of skin and nails	992	687	305	**2.25**	101	44	57	**0.77**	Infectious diseases	[64,65]*	Cryptic	N/A
Acute osteomyelitis	780	474	306	**1.55**	227	101	126	**0.8**	Musculoskeletal	[54]*	Cryptic	N/A
Encephalopathy, not elsewhere classified	1098	540	558	**0.97**	238	142	96	**1.48**	Neurological	[66]**	Cryptic	N/A
Chronic pain syndrome	1081	767	314	**2.44**	81	40	41	**0.98**	Neurological	[67]*	Cryptic	N/A
Elevated c-reactive protein (CRP)	569	355	214	**1.66**	104	45	59	**0.76**	Symptoms	[68,69]*	Cryptic	N/A

* Previously established association.

** Reported association.

*** Cryptic: not clear if it was a risk factor or a complication based on the data available in this study.

**** Clinical identifier: refers to a known symptom of SAI that is detected prior to confirmation of an SAI.

**Fig 2:**
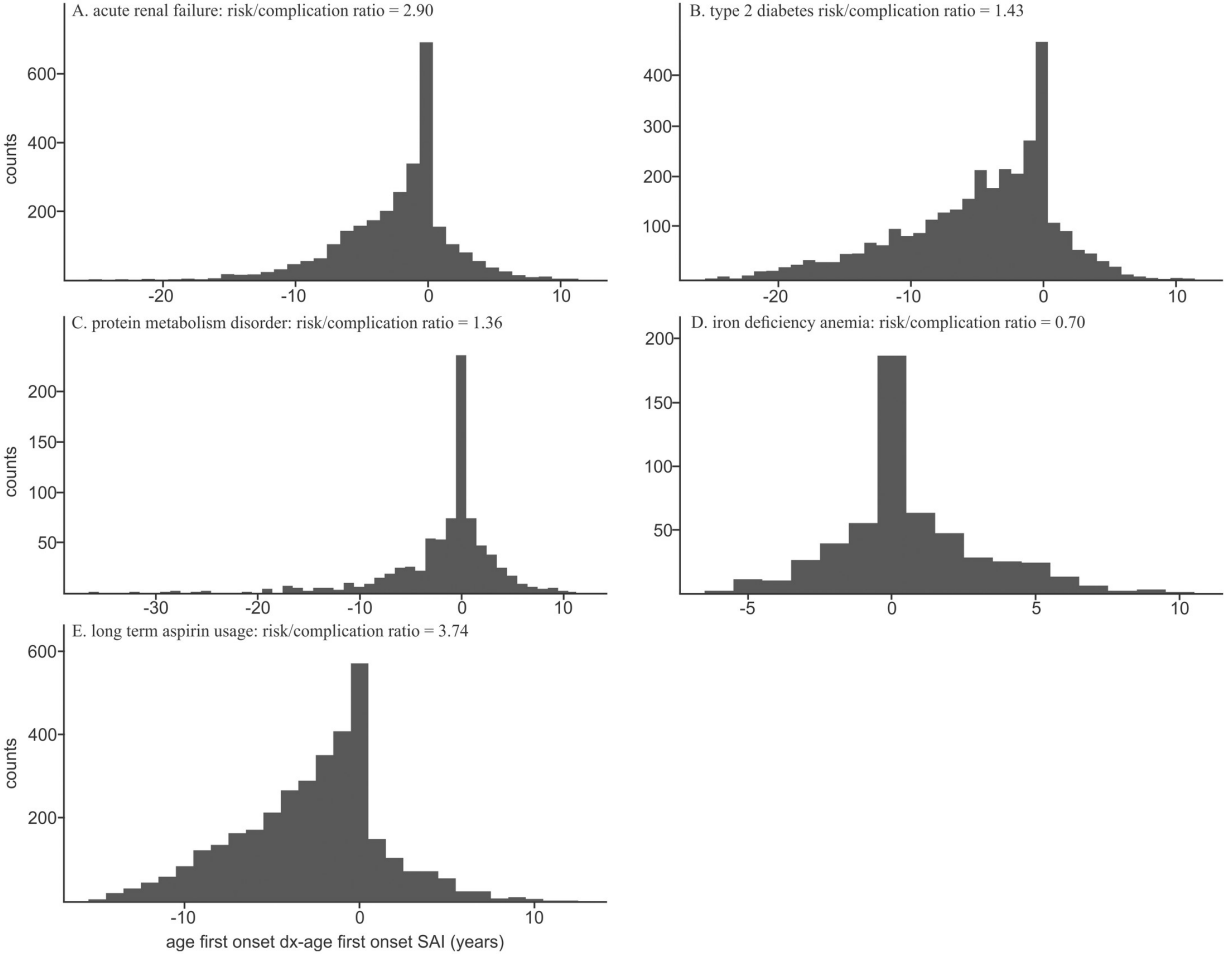
Histogram of known and novel associations. A) Acute renal failure (known association phecode 585.1). B) Type 2 diabetes (known association phecode 250.25). C) Plasma protein metabolism disorder (novel association, phecode 270.38). D) Aspirin usage (novel association, 457.3). E) Anemia of chronic disease (novel association, phecode 285.2).

Histograms for two known risk factors compared with our three new associations are displayed in Fig 2. The histogram counts represent the difference between the age of onset of phecode X and SAIs in one-year bins. One new risk factor, long-term aspirin usage, was mainly coded prior to the onset of SAI, similar to known risk factors such as T2D and acute renal failure (Fig 2A/B/E, and Table 2). Additionally, one association that was classified as a complication was novel (iron deficiency anemia due to chronic blood loss) and showed consistent trends with both lifelong and acute categories (Fig 2D and Table 2). Finally, anemia of chronic disease is classified as cryptic (Fig 2C and Table 2) because the lifelong ratio appears as a risk while acute as a complication.

### Novel associations are replicated in TriNetX and *All of Us*

**Fig 3:**
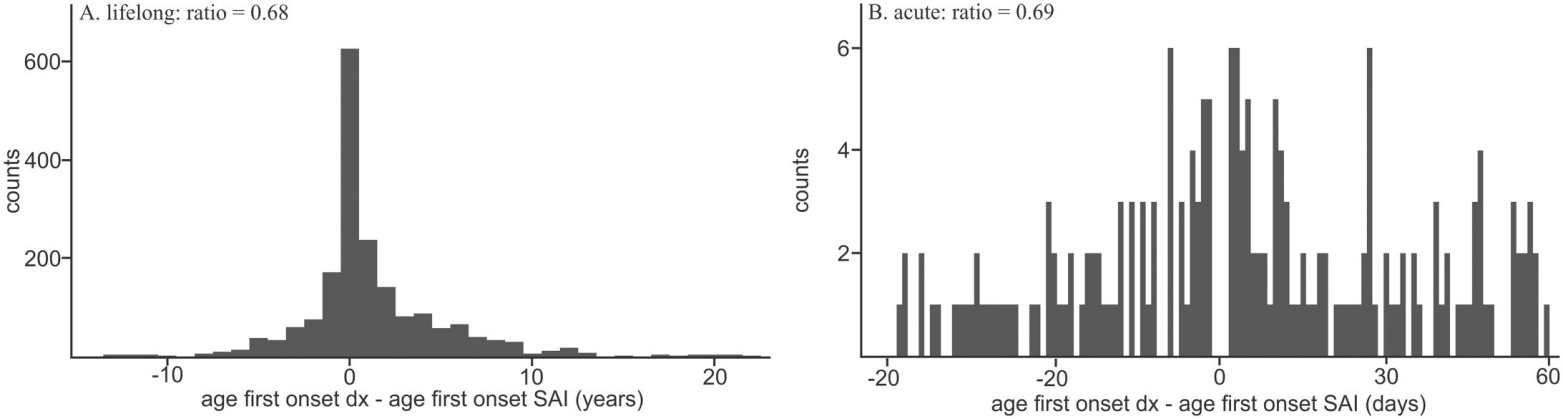
Histograms of difference between age of onset anemia of chronic disease and SAIs. TriNetX results after removal of patient at risk for SAIs. A) Lifelong count distribution across medical record. B) Acute count distribution over a window of +/- 60 days centered on age of onset of SAIs. Risk phecode include all diabetes, acute renal failure, all transplant, and chemotherapy codes. X axis measures the difference between the ages of first onset of phecode X—ages of first onset of SAIs. X axis is in years for A) and in days for B).

We verified the three novel associations in TriNetX and *All of Us* by calculating ORs using contingency tables. Inexplicably, ICD code for ‘long-term use of aspirin’ was not available in *All of Us* and as a result, we could not determine if the association between SAIs and long-term use of aspirin is replicable in *All of Us* or not. We additionally provided ORs corrected for possible confounding effects from known risk factors (chemotherapy, diabetes, organ transplants, surgeries, end-stage renal disease) (see S2 Table for phecode/ICD10 code usage). Correction was done by removing patients with these risk factors. We observed that ORs and statistical significance are generally maintained between MCHS, TriNetX, and *All of Us*, although they trended higher in TriNetX and lower in *All of Us* likely due to the sizes of the cohorts (Table 3). Interestingly, removing risk factors in the MCHS cohort had an impact on the anemia of chronic disease OR, wherein it decreased from 16.99 to 4.07 but remained highly significant. In contrast, OR for both anemia of chronic disease in the TriNetX and *All of Us* cohort remained constant (Table 3). Consistent with the replicated ORs, the age of first onset correlation also remained significant and mostly similar in effect size in TriNetX (Table 3). While in the MCHS cohort, anemia of chronic disease was found to be cryptic, even after correcting for at-risk patients, TriNetX showed this as a consistent complication of SAIs (Table 3 and Fig 3).

**Table 3:** Novel SAIs associations are replicated in the TriNetX and *All of US* cohort.

Cohort	Phenotype	Manual OR*			PheWAS Age of First Onset			Lifetime Onset Counts				Acute Onset Counts				Ratios Consistent?
		O.R.	S.E.	p-value	beta	S.E.	p-value	Total	Risk	Compl.	Ratio	Total	Risk	Compl.	Ratio	
MCHS all	iron deficiency anemia	8.43	0.43	3.32E-213	0.400	0.050	8.26E-13	541	223	318	0.70	110	42	68	0.62	yes—complication
MCHS no risk	iron deficiency anemia	7.34	0.75	7.64E-46	0.490	0.120	5.29E-05	123	49	74	0.66	28	13	15	0.87	yes—complication
TriNetX all	iron deficiency anemia	11.5	0.18	0.00E+00	0.407	0.018	5.30E-103	3049	1214	1835	0.66	401	160	241	0.66	yes—complication
TriNetX no risk	iron deficiency anemia	8.51	0.26	0.00E+00	0.422	0.035	5.43E-31	683	257	426	0.60	110	33	77	0.43	yes—complication
*AoU*RP all	iron deficiency anemia	4.32	1.13	5.14E-34	0.249	0.068	3.06E-4	233	106	127	0.83	18	11	7	1.57	no—cryptic
*AoU*RP no risk****	iron deficiency anemia	4.43	1.26	2.75E-10	n/a	n/a	n/a	n/a	n/a	n/a	n/a	n/a	n/a	n/a	n/a	
MCHS all	anemia of chronic disease	16.99	0.82	0.00E+00	0.220	0.010	1.72E-54	1710	1135	575	1.97	256	120	136	0.88	no—cryptic
MCHS no risk	anemia of chronic disease	4.07	0.18	6.67E-200	0.330	0.040	3.23E-18	307	201	106	1.90	60	27	33	0.82	no—cryptic
TriNetX all	anemia of chronic disease	25.34	0.32	0.00E+00	0.520	0.013	0.00E+00	6859	3267	3592	0.91	1040	490	550	0.89	yes—complication
TriNetX no risk	anemia of chronic disease	27.03	0.72	0.00E+00	0.657	0.035	2.19E-67	948	353	595	0.59	186	81	105	0.77	yes—complication
*AoU*RP all	anemia of chronic disease	11.81	1.09	2.36E195	0.382	0.039	1.91E-21	626	265	361	0.73	48	28	20	1.4	no—cryptic
*AoU*RP no risk	anemia of chronic disease	10.79	1.21	12.75E-33	0.585	0.111	2.16E-6	54	25	29	0.86		1	2	0.50	yes—complication
MCHS all	aspirin usage	5.25	0.16	0.00E+00	0.190	0.010	2.20E-61	3422	2700	722	3.74	298	185	113	1.64	yes—risk
MCHS no risk	aspirin usage	19.29	2.1	4.97E-70	0.190	0.020	5.28E-17	975	708	267	2.65	108	62	46	1.35	yes—risk
TriNetX all	aspirin usage	6.54	0.05	0.00E+00	0.421	0.016	5.81E-150	7163	3710	3453	1.07	803	497	306	1.62	yes—risk
TriNetX no risk	aspirin usage	4.86	0.06	0.00E+00	0.337	0.037	3.47E-19	1581	749	832	0.90	174	103	71	1.45	no—cryptic
*AoU*RP all***	aspirin usage	n/a	n/a	n/a	n/a	n/a	n/a	n/a	n/a	n/a	n/a	n/a	n/a	n/a	n/a	n/a
*AoU*RP no risk***	aspirin usage	n/a	n/a	n/a	n/a	n/a	n/a	n/a	n/a	n/a	n/a	n/a	n/a	n/a	n/a	n/a

* Odds Ratios (O.R.) calculated from a standard contingency table and therefore the results are not age and gender adjusted. This is due to data access restriction from TriNetX and the inability to perform logistic regression on the current TriNetX web platform.

** ’No risk’ refers to a cohort with patients with known risk factors removed. See Methods section 3 for risk factors. The ratio = counts of risk / counts of complication.

*** In the *All of Us* cohort, no ICD codes (ICD9:V58.67 and ICD10:Z79.82) linked to the "long-term use of aspirin" phecode 457.3 was identified.

**** Insufficient number of cases after removing individuals with risk factors.

S.E.: Standard Error

**Fig 4:**
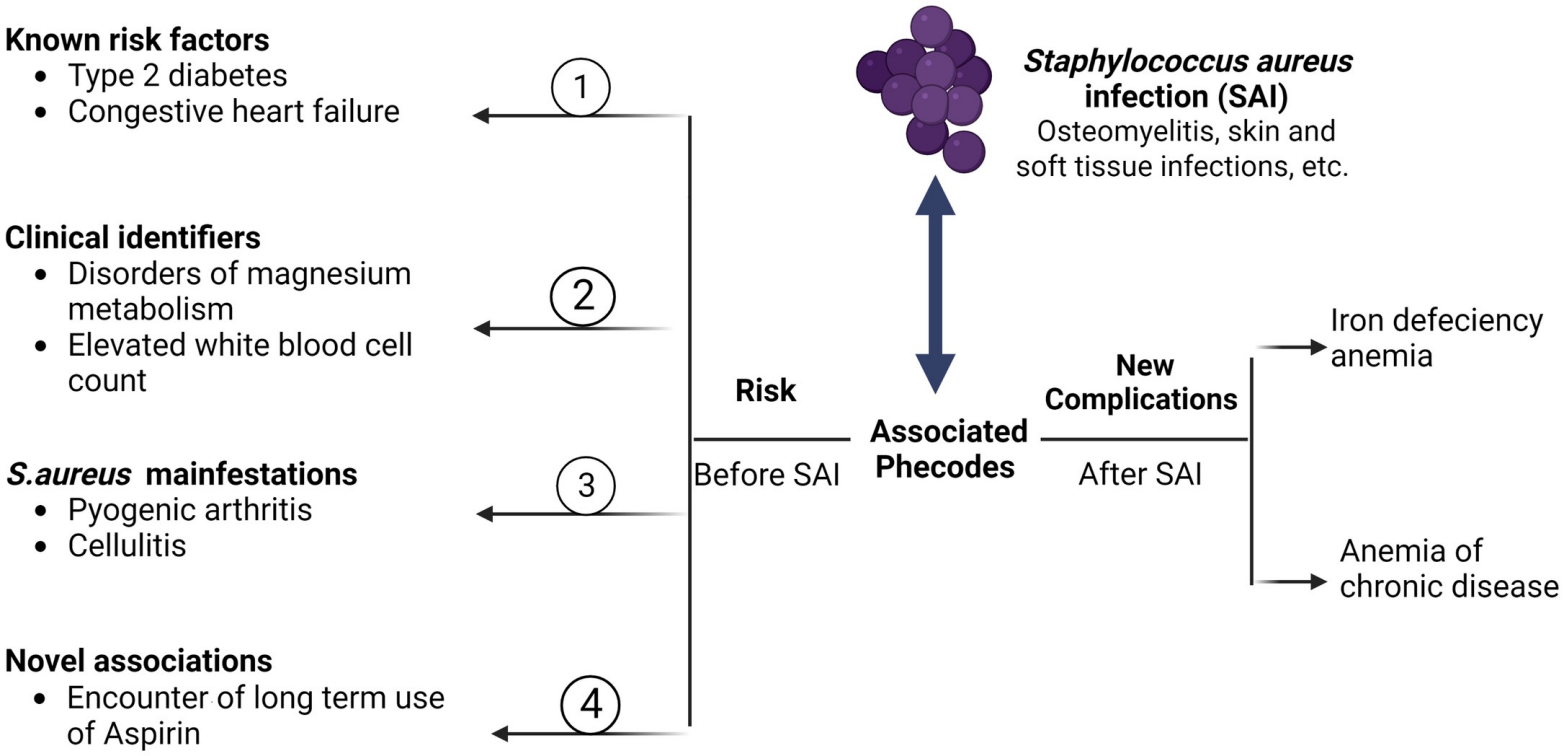
Graphical summary results of the PheWAS of SAIs. The left panel describes four risk factors categories with examples of two phecodes in each category: 1) Known Risk factors, 2) Clinical identifiers, 3) *S*. *aureus* manifestations, and 4) Novel Associations. The right panel describes two novel complications after SAIs.

Interestingly, the OR of the "long-term use of aspirin" observation in the MCHS increased to 19.29 from 5.25 after removing patients with known risk factors, whereas in TriNetX the OR decreased to 4.86 from 6.54. The observed cohort-specific trend appears to be due to differences in the frequency of known risk factors, which, according to the data, seem to be considerably higher in the MCHS cohort. Summary statistics for PheWAS of occurrence are shown in S3.2 Table in S2 File, and those of PheWAS of age correlation are shown in S4.2 Table in S3 File. Fig 4 summarizes our findings.

## Discussion

Today’s ease of access to the EHR systems of large health care organization and centralized EHR data set collection, such as TriNetX and *All of Us*, combined with sophisticated statistical screening methods, enable researchers to discover disease associations that were previously unachievable. In our study, we used a two-step PheWAS method to pinpoint and categorize health conditions not previously linked to SAIs. We found 41 unique health conditions associated with SAIs, grouped into 12 disease categories. The circulatory system had the largest representation with seven conditions, highlighting its significant role in the health complications caused by SAIs, regardless of the infection source. Three of the health conditions—diabetes, congestive heart failure, and acute renal failure—have already been associated with SAIs, validating our method [18,19]. To partially summarize our results, we classified the phenotypes associated with increased risk of SAIs into four main categories: i) previously identified known risk factors, ii) clinical identifiers, iii) novel associations, and iv) Staphylococcal manifestations. We define “clinical identifier” as a known symptom of SAI that is detected prior to confirmation of an SAI. The novel association category included: a) long-term use of aspirin, b) iron deficiency anemia, and c) anemia of chronic disease.

One advantage of our algorithm was in its ability to identify clinical identifiers as risk factors. For instance, previous studies have noted white blood cell count [20] or symptoms like delirium [21] after sepsis during patient evaluation. In our study, we recorded these indicators before the formal clinical confirmation of a SAI, indicating they can serve as warning signs for risk of infection. Additionally, our study supported the link between two previously suggested clinical identifiers and SAIs: disorders of magnesium metabolism (including both hypomagnesemia and hypermagnesemia) [22] and decubitus ulcers [23]. A deficiency of magnesium may reduce innate host defense against *S*. *aureus*, increasing the risk of infection. Notably, even after adjusting for diabetes or renal failure, magnesium imbalance remained a significant factor (see S3.2 Table in S2 File and S4.2 Table in S3 File). Xie and Yang (2016) highlighted the role of magnesium in fighting *S*. *aureus* infection, given its antimicrobial action against the bacterial membrane [22]. Decubitus ulcers, common in patients who are immobilized, can lead to various types of infections. It’s known that *S*. *aureus* can colonize these wounds [24], which may result in *S*. *aureus* bacteremia [23,25]. In line with this, we found cases where decubitus ulcer diagnosis was noted before an SAI incident. Identifying these kinds of associations is crucial, as it can contribute to the prevention and early diagnosis of SAIs, ultimately improving patient care.

Some conditions caused by *S*. *aureus*, referred to here as *S*. *aureus* manifestations, are typically identified around the time of SAIs diagnosis or shortly thereafter. These include diseases like cellulitis [26], MRSA pneumonia [27], carbuncle [26], and pyogenic arthritis [28] among others. There are also symptoms such as limb swelling, erythema, and edema that are coded before an SAI diagnosis. The seeming discrepancy may be due to the fact that identifying and confirming *S*. *aureus* involves a confirmation by lab-based culture, which can cause reporting delays [29].

In our study, long term use of aspirin was determined to be a risk factor for SAI. Known for its anticoagulant properties [30], aspirin use could heighten susceptibility to SAIs. Coagulation is a natural immune response to infection [31], so decreased coagulation might make an SAI more likely. It’s also possible that aspirin is prescribed to patients with abnormal coagulation. We suggest that the driver of SAIs is abnormal coagulation, rather than aspirin use per se, as these patients may already be on aspirin. Interestingly, aspirin use in hemodialysis patients has been reported to lower the risk of SAIs [32]. Alternatively, since SAI may also occur in patients who are chronically ill, aspirin usage may simply just be a marker of chronic illness specifically in patients with e.g., cardiac, vascular, or neurologic diseases as opposed to being directly related to SAI pathogenesis. In future focused studies, aspirin usage could be investigated by accounting for or excluding patients with confounding risk factors as mentioned above.

Our study verified two new associations, both of which were consistently observed in two additional cohorts, bringing the total to three cohorts confirming these novel complications. The first is iron deficiency anemia, which is physiologically linked to SAIs. *S*. *aureus* acquires iron from hemoglobin during invasive infections using its iron-regulated surface determinant receptor, IsdB [33]. This process aids in further invasion and persistence of *S*. *aureus* in the host, potentially leading to lower iron availability post-SAI. The second complication is anemia of chronic disease, previously reported as a risk factor for an SAI [9]. This condition may result from immune system changes affecting iron homeostasis due to bacterial, parasitic, fungal infections, or even cancer [34]. However, our findings suggest that anemia is more likely a complication than a risk factor. This is supported by Jensen et al., who reported the risk of anemia and hyponatremia following hospital-acquired *S*. *aureus* bacteremia [9]. Musher and colleagues also noted anemia preceding pneumococcal pneumonia, including severe cases with bacteremia [35]. Similarly, a mouse model showed *S*. *aureus* infection causing leukopenia, lymphopenia, neutrophilia, monocytosis, and microcytosis, the latter of which can lead to anemia [36]. Yet, to our knowledge, there are no existing reports specifying the type of anemia associated with SAIs. Importantly, for anemia of chronic disease, the long-term and acute risk/complication ratios remained low even after adjusting for known risk factors (lifetime 0.67, n = 2426; acute 0.69, n = 608). This suggests that anemia of chronic disease is likely a genuine complication of SAIs. Alternatively, as with long term usage of aspirin, anemia may just be a marker of chronic illness in patients who have previously coincidently been sick with SAI. Further studies will be needed to address this.

In our study, seventeen phecodes associated with SAIs were categorized as cryptic (Table 2) as they showed different lifetime and acute risk/complication ratios. For example, endocarditis is a disease that can result from SAIs in both community and hospital settings, making both associations plausible. For instance, patients might be hospitalized due to primary endocarditis [19] resulting from infections originating in the community (pre-SAI phecode). Alternatively, they might develop endocarditis following a *S*. *aureus* bacteremia acquired in the hospital (post-SAI phecode) [37].

Although our PheWAS approach has uncovered novel associations with SAIs, it is not without limitations. First and foremost, we intentionally designed our study to be strictly observational and not to determine cause and effect. As such, it should be regarded as only hypothesis generating. An important limitation of our study (and all studies based on ICD coding) is that there could be inconsistencies and variability in clinicians’ code selection. However, this limitation may be less relevant in our study due to the generalization of the phecode mapping system that clusters similar ICD-9 and ICD-10 codes to one phecode. Our phecode mapping system has its own distinct sets of limitations. One limitation inherent to the design of this study pertains to the simplification of the mapping ICD code to phecode mapping system. This is meant to simplify association testing, as close to 60,000 ICD-9 and ICD-10 codes exist. An immediate flaw of mapping several ICD codes is that it removes information from a treatment standpoint. However, this is not the focus of this study; rather, it is to identify risk factors/complications. Another weakness of the phecode mapping system that pertains to PheWAS of the age of onset is the inclusion of “history” codes. These codes provide an indication that a condition previously occurred with no mention of when or at what age. This could confound the statistics from the PheWAS of the age of first onset if the “history” ICD code occurs frequently. However, we find that, at least for SAI, the usage of the “history” code is infrequent, so it is not likely to significantly impact the results. Another weakness related the phecode system has to do with its categorization into disease categories that may not be fit from an infectious disease point of view. For example, cellulitis, abscess, and decubitus ulcer are all considered "dermatologic" but are different conditions. A similar mis-categorization along those lines is observed with osteomyelitis, MRSA pneumonia, and endocarditis, which are listed under musculoskeletal, respiratory, and circulatory systems, respectively, but not infectious. However, the key point in this disease category is that it reflects how organ systems are affected, and therefore, ones that are more prone to infections. One limitation of our study is the method we used to classify associations as either risk factors or complications. This classification should be considered within the context of the already understood pathophysiology of the disease. Sometimes a complication may appear as a risk factor due to the timing of phenotype or clinical identifier observation (e.g., cellulitis and increased white blood cell count) and coding in EHR. A fourth limitation concerns the generalization of our results to distinct ancestries. Most of the patients in the MCHS are of European ancestry and many have no reported ethnicity but are presumably European given known regional history. Our results are thus not generalizable to all ancestry but reflects that of white/European.

In conclusion, we have developed a unique PheWAS strategy to uncover a range of associations between various phenotypes and SAIs. Our study offers a comprehensive hypotheses catalogue of phenotypes associated with SAIs, establishing a foundation for future SAI research that will hopefully benefit SAI prevention and treatment.

## Supporting information

S1 Data (XLSX)

S1 File (XLSX)

S2 File (XLSX)

S3 File (XLSX)

S1 Table **S1.1 Table**. **Cohort demographics for PheWAS of phecode occurrences. S1.2 Table**. **Cohort demographics for PheWAS of phecode age of first onset**.
(XLSX)

S2 Table Statistical models and SAI risk factors used. (XLSX)

S3 Table **S3.1 Table. PheWAS results for SAI occurrences Vs phecode occurrences**. Basic. **S3.2 Table. PheWAS results for SAI occurrences Vs phecode occurrences**. After removing patient with known SAI risk factors (only sentinel phecode where screened).
(XLSX)

S4 Table **S4.1 Table. PheWAS results for SAI age of first onset Vs phecode age of first onset**. Basic. **S4.2 Table. PheWAS results for SAI age of first onset Vs phecode age of first onset**. After removing patient with known SAI risk factors (only sentinel phecode where screened).
(XLSX)

S5 Table Sentinel phecode selection. (XLSX)

S6 Table Classification of sentinel phecodes into disease categories. (XLSX)
